# Mendelian randomization study and mediation analysis about the relation of inflammatory bowel disease and diabetic retinopathy: the further exploration of gut-retina axis

**DOI:** 10.3389/fendo.2024.1382777

**Published:** 2024-06-14

**Authors:** Jiayi Lin, Yaqi Cheng, Simin Gu, Siqi Song, Huini Zhang, Jianbing Li, Shiqi Ling

**Affiliations:** Department of Ophthalmology, The Third Affiliated Hospital of Sun Yat-sen University, Sun Yat-sen University, Guangzhou, China

**Keywords:** gut-retina axis, inflammatory bowel disease, diabetic retinopathy, Mendelian randomization, mediation analysis

## Abstract

**Background:**

The concept of the gut-retinal axis proposed by previous scholars primarily focused on the relationship between intestinal microbiota and retinal diseases, and few further expanded the relationship between intestinal diseases and retinal diseases. To further substantiate the concept of the gut-retinal axis, we analyzed inflammatory bowel disease (IBD) and diabetic retinopathy (DR) using Mendelian randomization (MR), and use mediation analysis to further explore the potential substances that influence this causal relationship.

**Methods:**

The genome-wide association study’s (GWAS) summary statistics for genetic variations were utilized in a Mendelian randomization (MR) investigation. GWAS data on IBD (including ulcerative colitis (UC), Crohn’s disease (CD), and IBD) for non-Finnish Europeans (NFE) were sourced from published articles. In contrast, data on DR (including DR and diabetic maculopathy (DMP)) were obtained from FinnGen R9. The causal relationship has been investigated using inverse variance weighted (IVW), MR-Egger, and weighted median and sensitivity analysis was applied to verify the stability of the results. In addition, we applied mediation analysis to investigate whether circulating inflammatory proteins and plasma lipids played a mediating role, and calculated its effect ratio.

**Results:**

The causal relationship between IBD and DR was discovered by employing the inverse variance weighted (IVW) method and weighted median method. In forward MR, UC was significantly associated with lower risk of DR (IVW: OR=0.874; 95%CI= 0.835–0.916; P value= 1.28E-08) (Weighted median: OR=0.893; 95%CI= 0.837–0.954; P value= 7.40E-04). In reverse MR, it was shown that DR (IVW: OR=0.870; 95%CI= 0.828–0.914; P value= 2.79E-08)(Weighted median: OR=0.857; 95%CI= 0.801–0.916; P value= 6.40E-06) and DMP (IVW: OR=0.900; 95%CI= 0.865–0.937; P value= 3.34E-07)(Weighted median: OR=0.882; 95%CI= 0.841–0.924; P value= 1.82E-07) could reduce the risk of CD. What’s more, DR is associated with a lower risk of IBD according to genetic prediction (IVW: OR=0.922; 95%CI= 0.873–0.972; P value= 0.002) (Weighted median: OR=0.924; 95%CI= 0.861–0.992; P value= 0.029). Fibroblast growth factor 21 (FGF21), phosphatidylcholine (PC), and triacylglycerol (TG) serve as mediators in these relationships.

**Conclusions:**

Our research offers novel insights and sources for investigating the gut-retina axis in the genetic relationship between IBD and DR. We discover four mediators and more about the association between the intestine and retinal disorders and provide more evidence for the gut-retinal axis theory.

## 1 Introduction

During the past few years, there has been a significant global surge in the incidence of inflammatory bowel disease (IBD), which consists of Crohn’s disease (CD), ulcerative colitis (UC), and indeterminate colitis. IBD is a chronic condition with a predilection for children and young adults, and it is linked to a range of adverse outcomes such as cancer, hospitalizations, surgical interventions, and infections (1). The pathogenesis of IBD is intricate and multifactorial, involving intricate interactions between genetic susceptibility, environmental triggers, immune-mediated mechanisms, and microbial influences. Extensive research efforts have been dedicated to unraveling the complexities of this disease. The management of IBD aims to achieve clinical remission through a combination of dietary interventions and pharmacotherapy targeting inflammation. This comprehensive approach enhances the overall quality of life for individuals afflicted by IBD (2).

Diabetic retinopathy (DR) is a noticeable ocular manifestation of diabetes mellitus (DM), affecting approximately 30 to 40% of individuals with diabetes (3). Hyperglycemia can cause capillary occlusion and an increase in vascular permeability, which can result in nonproliferative DR (NPDR). A proliferative phase of DR (PDR), typified by the creation of new blood vessels, may occur after this phase (4). Among the various complications of DR, diabetic maculopathy (DMP) is a prominent cause of legal blindness, primarily characterized by diabetic macular edema (DME). DME occurs when fluid builds up in the macula, leading to visual impairment. Adequate assessment, screening, and imaging techniques can aid in preventing vision loss associated with DR. However, despite these measures, DR still imposes a substantial societal burden, as individuals cope with increased life pressures and potentially modify their dietary habits (5, 6).

Currently, numerous studies have demonstrated a connection between the metabolism of intestinal microorganisms and retinal diseases, so the concept of the gut-retinal axis has been proposed by scholars (7). The gut microbiota has been found to influence the expansion of various retinal disorders, including diabetic retinopathy, optic neuritis, age-related macular degeneration, and retinopathy of prematurity. As a result, controlling the gut microbiota has grown into a potential approach for treating or preventing such eye disorders (8). However, most studies investigating the gut-retinal axis have primarily focused on the field of intestinal microorganisms, whereas the relationship between intestinal diseases and retinal diseases remains largely unclear. The connection between intestinal and retinal diseases has been studied through observational research, but the findings have been limited. Uveitis associated with IBD is well-documented, but there have been few studies investigating the link between IBD and DR. Both IBD and DR involve barrier dysfunction. In IBD, large amounts of tissue inhibitors of metalloproteases (TIMP)-free matrix metalloproteases (MMPs) are produced by neutrophils, while in DR, mononuclear leukocytes synthesize MMP-9 in a balanced manner with TIMP-1. Therefore, MMP-9 may be a therapeutic target for both conditions (9). Additionally, APE1/Ref-1, a multifunctional signal transduction enzyme, is implicated in the pathogenesis of both IBD and DR (10). To further explore the gut-retina axis, we have chosen IBD and DR as research subjects due to their close association with metabolism and inflammatory factors.

In randomized controlled trials, there are always issues with the viability, logic, and safety of investigation. The conclusions obtained from individual randomized controlled trials are potentially biased by uncontrolled confounding factors. A statistical technique known as Mendelian randomization (MR) can be employed to overcome these limitations. MR prevents reverse causation and facilitates the derivation of valid conclusions regarding the causal relationship between exposure and outcome variables. This approach utilizes single-nucleotide polymorphisms (SNPs) as instrumental variables (IVs) to assess the causal association between two phenotypes (11). The alleles of the genetic variant associated with the exposure of target phenotype are randomly assigned and not susceptible to reverse causation (12, 13). In our study, we utilized genotype-phenotype associations from publicly available Genome-wide association studies (GWASs) in a two-sample bidirectional Mendelian randomization investigation to examine whether causal link exists between IBD and DR.

## 2 Materials and methods

### 2.1 Study design

**Figure 1:**
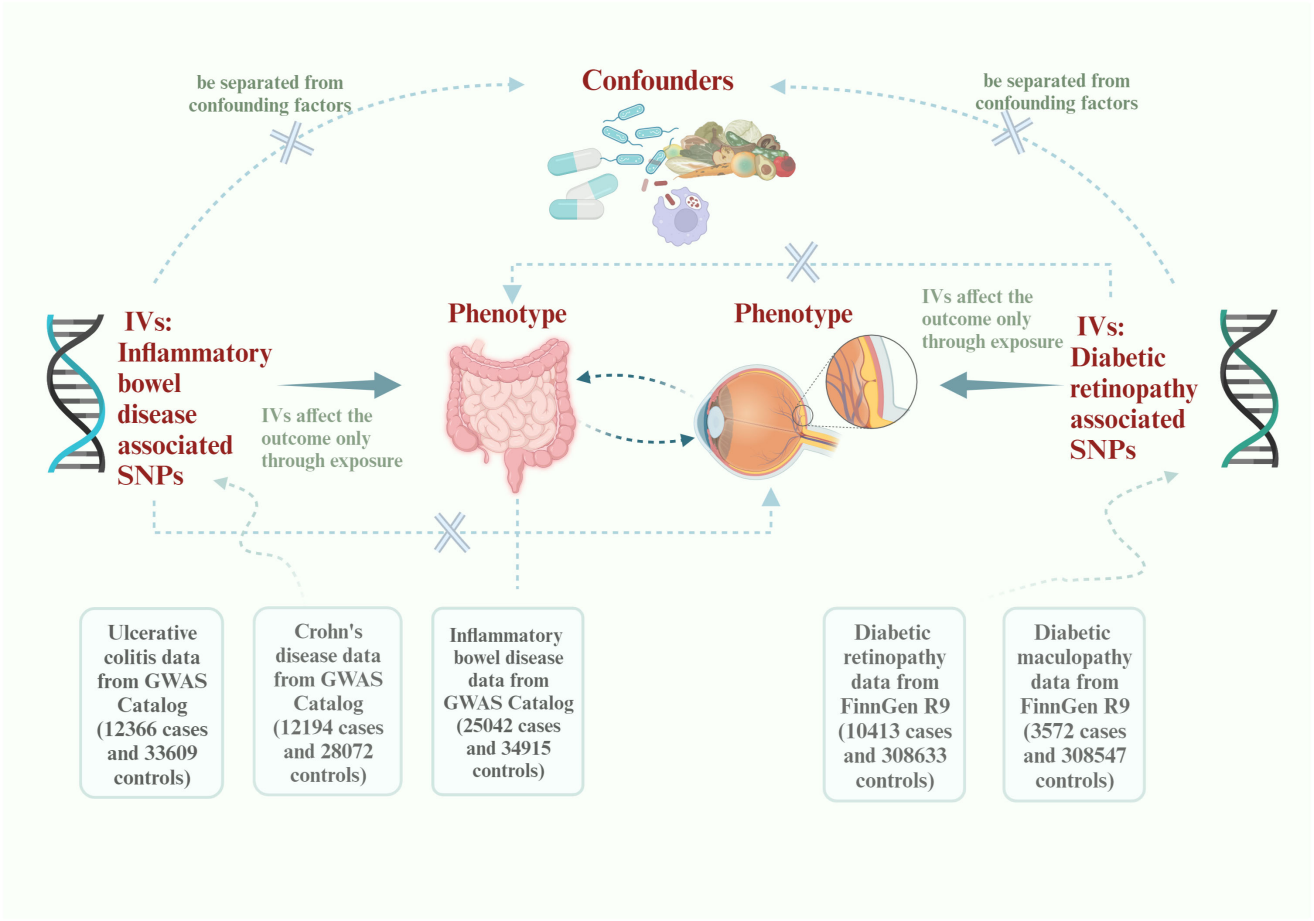
Flowchart of this Mendelian randomization study. IVs, instrumental variables; SNPs, single-nucleotide polymorphisms; GWAS, Genome-wide association study.

A brief description of this bidirectional MR design between IBD and DR is displayed in **Figure 1**. The data about the IBD could be extracted from the study of non-Finnish European (NFE) published in *Nature genetics* by Katrina (14, 15). The data about DR could be divided into DR and diabetic maculopathy, an important subcategory. All data come from published articles or publicly available GWAS statistics. With bidirectional MR, IBD is viewed as the exposure and DR as the result in forward MR analysis. In contrast, DR is considered as the exposure and IBD as the result in reverse MR analyses (16). Due to the limited size of SNPs of DMP, we could only use this data in reverse MR analysis. After conducting a bidirectional Mendelian randomization analysis, we proceeded with a mediation analysis to further investigate the mechanisms underlying the interaction between IBD and DR. The whole study is based on three basics instrumental variable (IV) assumptions: I The chosen instrumental variables should have a substantial correlation with exposure. II The chosen instrumental variables should be separated from confounding factors. III The chosen instrumental variables must affect the outcome only through exposure. Research may only be conducted following those as mentioned above three fundamental principles.

### 2.2 Data source of exposures, outcomes and mediators

The GWAS statistics on IBD and its two subtypes, UC and CD, come from the GWAS study published in *Nature genetics* (14). The study subjects were European populations not included in the whole-genome meta-analysis, referred to as non-Finnish European (NFE) (15). The study involved 12,366 UC cases, 12,194 CD cases, and 25,042 IBD cases. This data is available from the GWAS catalog (https://www.ebi.ac.uk/gwas/publications/28067908) and the IEU open GWAS database (https://gwas.mrcieu.ac.uk/). The GWAS statistics of DR were collected from the FinnGen R9 research consortium (https://r9.finngen.fi/pheno/DM_RETINOPATHY_EXMORE). This data included 10413 DR cases and 308633 controls of Finnish ancestry. The definition of DR in our statistics is a chronic, pathological complication associated with diabetes mellitus (DM), where retinal damages are incurred due to microaneurysms in the vasculature of the retina. In addition, the GWAS of DMP was used in our analysis because it’s an important sub-disease of DR. This GWAS was also extracted from the FinnGen R9 research consortium (https://r9.finngen.fi/pheno/DM_MACULOPATHY_EXMORE) which encompassed 3572 cases and 308547 controls. DMP is recognized as a loss of vision in the central portion of the retina (macula) caused by DM.

The relationship between IBD and DR and their association with the body’s inflammatory state and lipid composition is currently recognized (17). We conducted a study to investigate if circulating inflammatory proteins and plasma lipids play a role in mediating the relationship between IBD and DR. *Zhao* conducted a genome-wide study on 91 plasma proteins (GCST90274758 - GCST90274848) (18), and we utilized this data for our research. Additionally, *Ottensmann* carried out a genome-wide analysis on 179 lipids from 13 lipid classes (GCST90277238 - GCST90277416) (19). The data is available from the GWAS catalog (https://www.ebi.ac.uk/gwas/home).

### 2.3 Ethics statement

The data used in this study to analyze the relationship between IBD and DR can be obtained from public literature or databases. The institutional ethical committees of each GWASs utilized in this study confirmed their approval. No further ethical approval is required in this situation.

### 2.4 Selection of genetic instrumental variables

At first, we performed forward MR analysis, and UC, CD and IBD were chosen for the exposure variable. Firstly, a subset of SNPs was chosen as IVs based on their significance level below the genome-wide threshold of 5×10^−8^, respectively. Secondly, we implemented a linkage disequilibrium (LD) analysis based on European-based 1,000 Genome Projects (R2 <0.001, clumping distance = 10,000 kb) to meet the MR core assumptions. The SNPs that failed to satisfy the requirements were deleted. Thirdly, Each SNP’s efficacy as an IV was determined using the F-statistic: a value above ten suggested a strong instrument. Finally, we removed all palindromic sequences to ensure the selected SNPs were referred to the same allele when harmonizing the effects of the SNPs on exposure and outcome. We corrected the strand for SNPs with different effect alleles, guaranteeing the effect allele was the same in both databases.

In reverse MR analysis, the DR and DMP were selected for the exposure variable. Considering the limited size of IVs obtained from the GWAS of DMP, we selected the wide significance (P<5×10^−6^) as the threshold to get more SNPs. The settings for the parameters and leftover flow were the same as in the forward MR.

In the mediation analysis, 91 circulating inflammatory proteins and 171 plasma lipid components were considered as potential mediators. Due to the limited number of IVs, we also relaxed the selection threshold (P<5×10^−6^) to include more SNPs.

### 2.5 Mendelian randomization analysis

The primary approach for estimating the causal effect values unbiasedly was the inverse variance weighted (IVW) test, which prevented confounding factors in the absence of a horizontal pleiotropy. IVW is the most widely used method combining the Wald ratio in fixed-effect meta-analysis to infer the presence and the strength of the causal effect between an exposure and an outcome (20). At the same time, we highlighted the significance of the weighted median method because it can maintain consistency where invalid instrumental variables account for as much as 50% of the data (21). By combining the results obtained by these two methods, we can improve the solidity of the final results. Furthermore, MR-Egger regression was performed to estimate the causal effect adjusted for directional pleiotropy. It has the lowest capability so its results should be cautiously treated. When only one SNP passed quality control, causal associations were evaluated using the Wald ratio (WR) method.

### 2.6 Sensitivity analysis

Several sensitivity analyses were applied to detect the horizontal pleiotropy and heterogeneity, which may improve the reliability of results. Cochran’s Q test, heterogeneity, the leave-one-out method, RadialMR and MR PRESSO each play a special role in a sensitivity analysis. Finding the heterogeneity is essential since it helps decide when it’s suitable to combine all individual outcomes into a single summary measure (22). Cochran’s Q test is the main method to evaluate heterogeneity between different samples. Since we consider the outcomes of our study to be stable and reliable in the absence of heterogeneity, the test’s P value ought to be greater than 0.05. We are more concerned about horizontal pleiotropy since it defies fundamental MR analysis assumptions (the chosen instrumental variables must impact the outcome only through exposure). MR-Egger intercept was applied to assess the presence of horizontal pleiotropy between the IVs and the result, while MR PRESSO global test also detected horizontal pleiotropy. According to statistical presumptions, we cannot conclude that the study shows horizontal pleiotropy when the P value>0.05. The leave-one-out method detected whether results change due to a certain SNP. This is another efficient way to guarantee the outcome’s stability. RadialMR was applied to exclude the outlier SNPs because methods for removing outliers can successfully lessen bias in MR estimates.

The Bonferroni method was adopted to correct all P values. Calculating Bonferroni’s adjustment involves dividing the total number of tests by the alpha value. In forward MR analysis, if the corrected P value <0.016, we have more grounds for believing that exposure and result are strongly correlated. If the adjusted P value is less than 0.05 but greater than 0.016, we may consider the possibility of an exposure-outcome relationship into account. As with forward MR, we may be more certain that the exposure is highly correlated with the result in reverse MR when the P value is smaller than 0.0083. P values between 0.0083 and 0.05 indicate a possible association between the two factors.

### 2.7 Mediation analysis linking IBD with DR via circulating inflammatory proteins and plasma lipids

We conducted a mediation analysis to connect IBD with diabetic retinopathy DR through circulating inflammatory proteins and plasma lipids. After performing a bidirectional Mendelian randomization analysis, we found positive associations between exposure factor A1 and outcome factor B1. Next, we investigated the correlations between 91 circulating inflammatory proteins and 179 plasma lipids with the outcome factor B1. We selected the substances that showed positive associations for further analysis. These selected substances were then analyzed to determine their correlation with exposure factor A1 when considered as outcome factors B2. When intermediary substances were found to be correlated with both exposure and outcome factors, we calculated the proportion of the mediating effect attributable to each intermediary substance using delta method (23).

We use RStudio (2024.04.1 + 748) to perform the above process, and with the help of TwoSampleMR R packages (version 0.5.7).

## 3 Results

### 3.1 Causal effect in forward MR analysis

**Figure 2:**
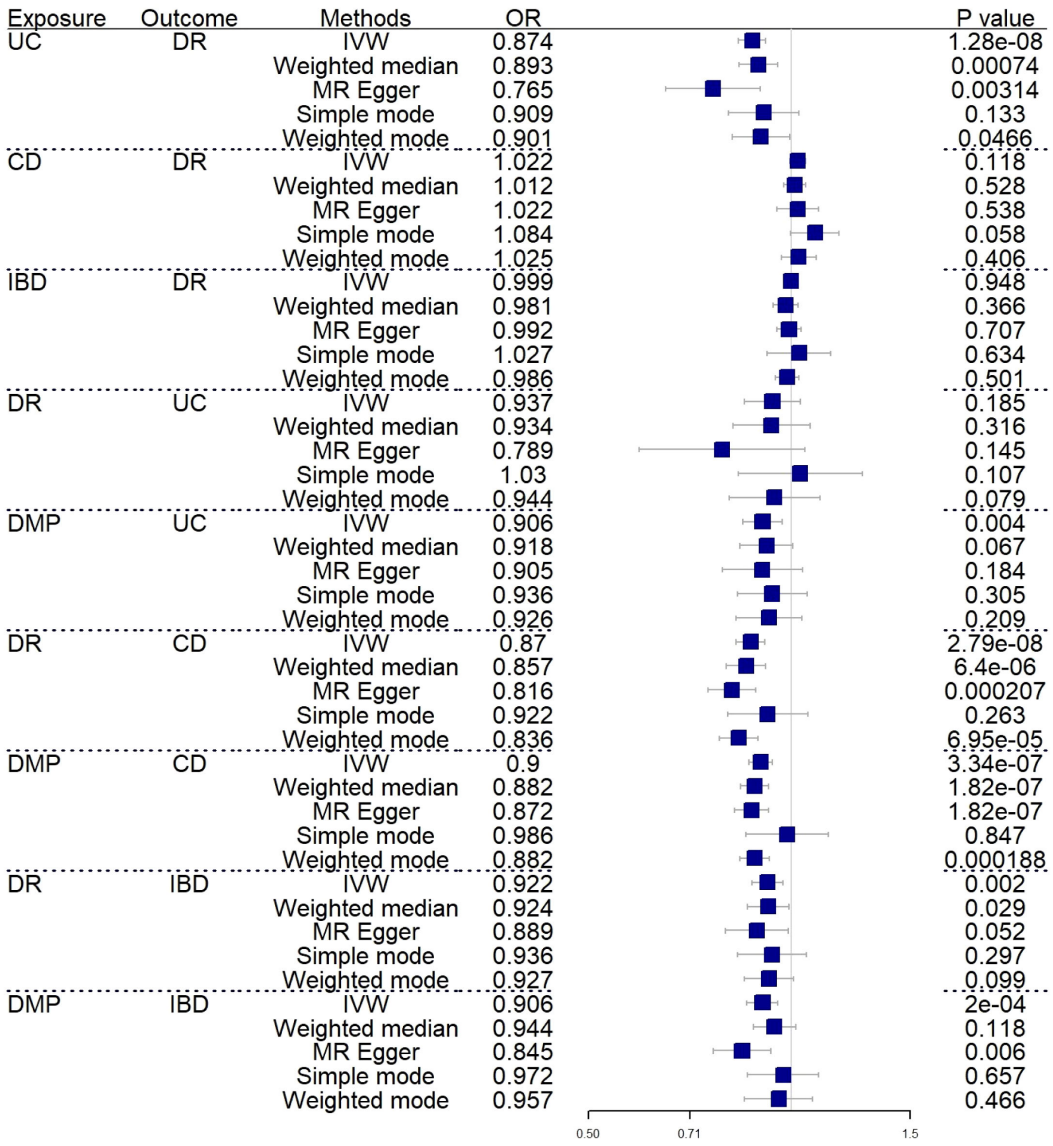
Forest plot of MR analysis results between inflammatory bowel disease and diabetic retinopathy. UC, ulcerative colitis; CD, Crohn’s disease; IBD, inflammatory bowel disease; DR, diabetic retinopathy; DMP, diabetic maculopathy; IVW, inverse variance weighted; OR, odds ratio.

A forward MR analysis was conducted to examine the IBD and its two subtypes, whether about DR. The figure shows that IBD did not show a genetic relationship with DR. However, the results of two subtypes are not fully aligned with IBD (IVW: OR=0.999; 95%CI= 0.974–1.024; P value= 0.948) (Weighted median: OR=0.981; 95%CI= 0.939–1.023; P value= 0.366). UC was negatively correlated with DR (IVW: OR=0.874; 95%CI= 0.835–0.916; P value= 1.28E-08) (Weighted median: OR=0.893; 95%CI= 0.837–0.954; P value= 7.40E-04), CD did not show a causal relationship with DR as well (IVW: OR=1.022; 95%CI= 0.994–1.049; P value= 0.118) (Weighted median: OR=1.012; 95%CI= 0.974–1.051; P value= 0.538). **Supplementary Material 1** shows the complete results and **Table 1** shows the sensitivity analysis results. **Figure 2** shows the forest plot of the analysis results.

**Table 1:** Heterogeneity and pleiotropy analysis of inflammatory bowel disease with diabetic retinopathy.

Exposure	Outcome	Methods	Q	P (heterogeneity)	P (Egger intercept)	MR-PRESSO global test P value
UC	DR	IVW	27.52	0.383		
		MR Egger	24.61	0.484	0.101	0.393
CD	DR	IVW	69.23	0.170		
		MR Egger	69.23	0.148	0.986	0.227
IBD	DR	IVW	79.59	0.523		
		MR Egger	79.41	0.497	0.673	0.567
DR	UC	IVW	10.03	0.528		
		MR Egger	8.43	0.587	0.235	0.648
DMP	UC	IVW	9.76	0.462		
		MR Egger	9.76	0.552	0.975	0.616
DR	CD	IVW	13.55	0.632		
		MR Egger	9.92	0.825	0.076	0.652
DMP	CD	IVW	15.78	0.397		
		MR Egger	13.62	0.478	0.164	0.370
DR	IBD	IVW	8.01	0.843		
		MR Egger	7.44	0.827	0.462	0.920
DMP	IBD	IVW	13.82	0.312		
		MR Egger	11.18	0.428	0.136	0.299

MR, Mendelian Randomization; UC, Ulcerative colitis; CD, Crohn’s disease; IBD, Inflammatory bowel disease; DR, Diabetic retinopathy; DMP, Diabetic maculopathy; IVW, Inverse variance weighted.

**Figure 3:**
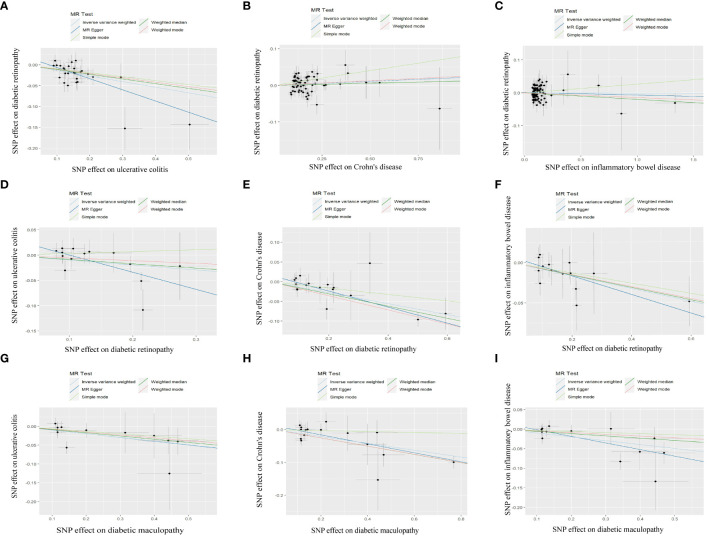
Scatter plot of MR analysis results: **(A)** scatter plot of MR analysis results between UC and DR; **(B)** scatter plot of MR analysis results between CD and DR; **(C)** scatter plot of MR analysis results between IBD and DR; **(D)** scatter plot of MR analysis results between DR and UC; **(E)** scatter plot of MR analysis results between DR and CD; **(F)** scatter plot of MR analysis results between DR and IBD; **(G)** scatter plot of MR analysis results between DMP and UC; **(H)** scatter plot of MR analysis results between DMP and CD; **(I)** scatter plot of MR analysis results between DMP and IBD. UC, ulcerative colitis; CD, Crohn’s disease; IBD, inflammatory bowel disease; DR, diabetic retinopathy; DMP, diabetic maculopathy.

**Figure 4:**
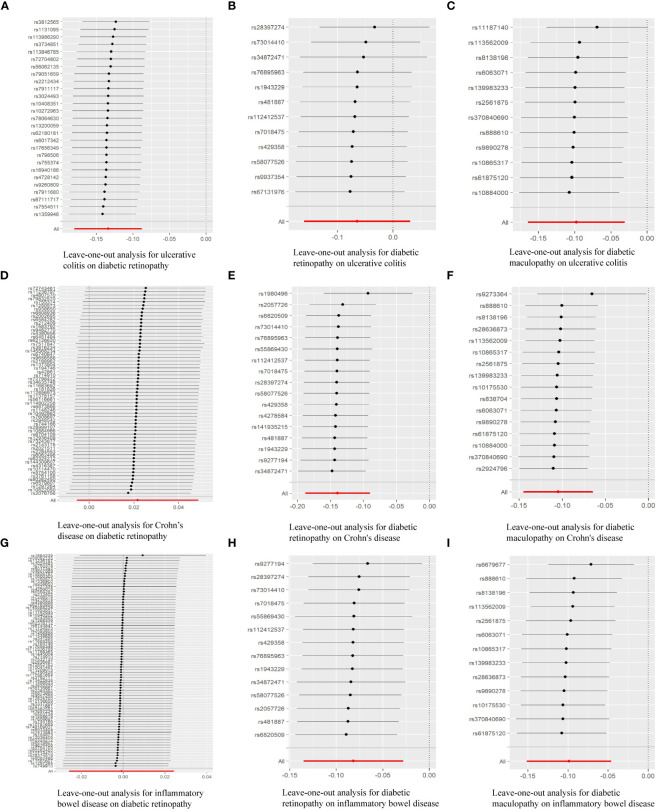
Leave-one-out analysis of the effect of individual SNPs of MR analysis results: **(A)** Leave-one-out analysis for ulcerative colitis on diabetic retinopathy; **(B)** Leave-one-out analysis for diabetic retinopathy on ulcerative colitis; **(C)** Leave-one-out analysis for diabetic maculopathy on ulcerative colitis; **(D)** Leave-one-out analysis for Crohn’s disease on diabetic retinopathy; **(E)** Leave-one-out analysis for diabetic retinopathy on Crohn’s disease; **(F)** Leave-one-out analysis for diabetic maculopathy on Crohn’s disease; **(G)** Leave-one-out analysis for inflammatory bowel disease on diabetic retinopathy; **(H)** Leave-one-out analysis for diabetic retinopathy on inflammatory bowel disease; **(I)** Leave-one-out analysis for diabetic maculopathy on inflammatory bowel disease.

We rigorously conduct sensitivity analysis to guarantee the integrity of the findings. The results of UC had passed various tests. The p-value in the heterogeneity test was 0.383, and the p-value in the Egger-intercept test was 0.101. scatter plots and Leave-one-out plots are presented in **Figures 3**, **4**. After applying the Bonferroni correction, the relationship between UC and DR was still statistically significant.

### 3.2 Causal effect in reverse MR analysis

In reverse MR analysis, we respectively investigated the possibility that DR and DMP are genetically related to IBD and its subtypes. To find possible correlations between DMP and IBD, we used a lenient P-value (P<5E-06). As shown in **Figure 2**, the results indicated that DR (IVW: OR=0.870; 95%CI= 0.828–0.914; P value= 2.79E-08)(Weighted median: OR=0.857; 95%CI= 0.801–0.916; P value= 6.40E-06) and DMP (IVW: OR=0.900; 95%CI= 0.865–0.937; P value= 3.34E-07)(Weighted median: OR=0.882; 95%CI= 0.841–0.924; P value= 1.82E-07) had causal relationships with the risk of CD. It is genetically predicted that DR is about the risk IBD (IVW: OR=0.922; 95%CI= 0.873–0.972; P value= 0.002) (Weighted median: OR=0.924; 95%CI= 0.861–0.992; P value= 0.029). What’s more, the IVW results indicated DMP is about the UC (IVW: OR=0.906; 95%CI= 0.848–0.969; P value= 0.004) and the IBD (IVW: OR=0.906; 95%CI= 0.859–0.955; P value= 0.0002). Only one analysis, IVW, showed a positive relationship between DMP and UC/IBD, so we have reservations about the positive results. Sensitivity analysis revealed no notable heterogeneity and horizontal pleiotropy.

### 3.3 Result of mediation analysis

**Figure 5:**
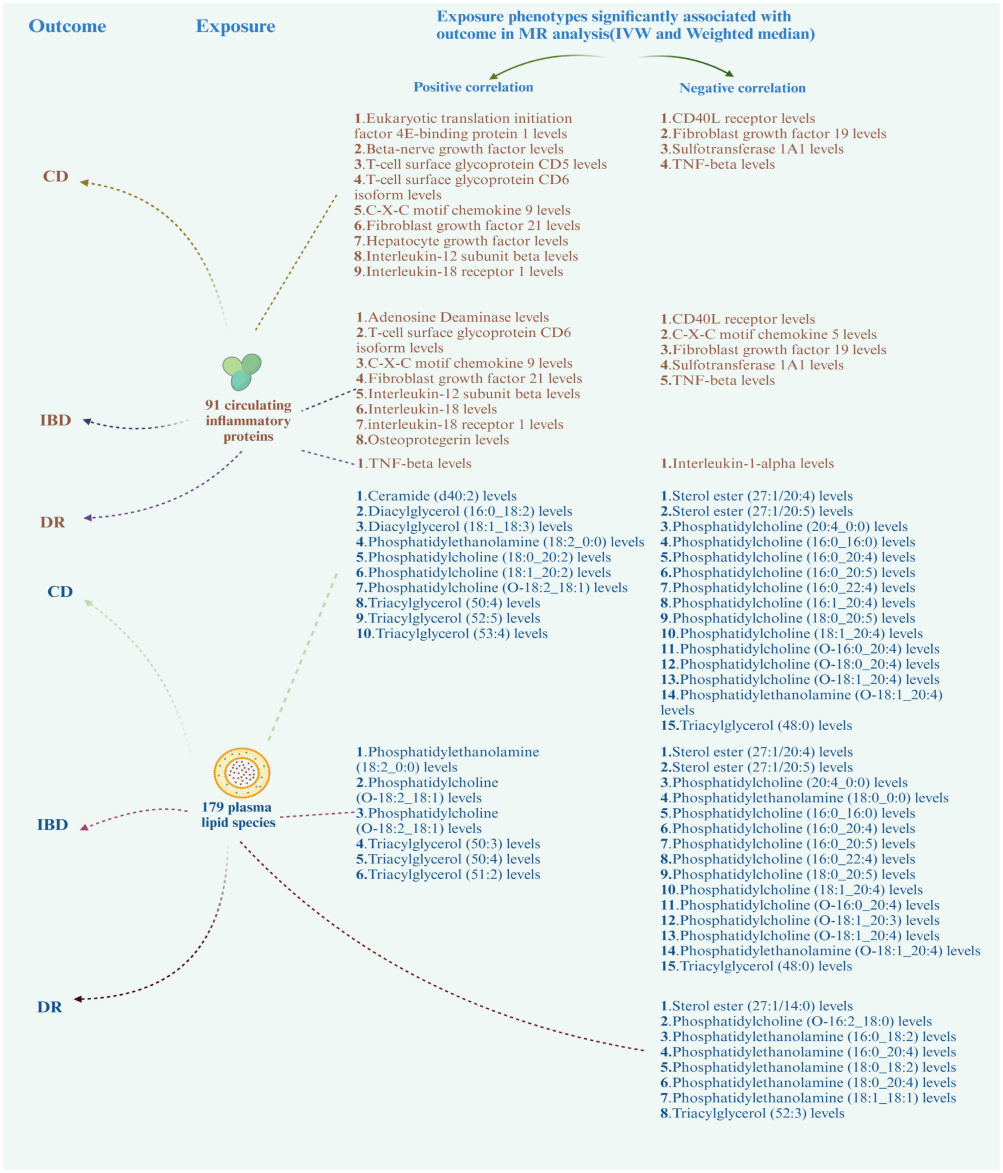
Correlation of circulating inflammatory proteins and plasma lipids with CD, IBD, and DR. CD, Crohn’s disease; IBD, inflammatory bowel disease; DR, diabetic retinopathy.

Based on the positive results mentioned earlier, we initially used the MR method to analyze the connection between 91 circulating inflammatory proteins and 171 plasma lipids with CD (for more information, please refer to **Supplementary Materials S2**, **S6**). We identified that a total of 13 circulating inflammatory proteins were associated with CD, with 9 proteins positively correlated and 4 proteins negatively correlated. Additionally, 25 plasma lipids were found to be associated with CD, with 10 lipids showing a positive correlation and 15 lipids showing a negative correlation. We then analyzed the correlation between these 13 circulating inflammatory proteins and 25 plasma lipids as the outcome factor with DR and DME as the exposure factor. We also calculated the mediating effect ratios. Similarly, we analyzed the association between the 91 circulating inflammatory proteins and 171 plasma lipids with IBD and DR, respectively (for more information, please refer to **Supplementary Materials S3**–**S5**, **S7**). Relevant results are shown in **Figure 5**; **Table 2**. After completing these steps, we identified Fibroblast Growth Factor 21, Phosphatidylcholine (20:4_0:0), and Phosphatidylcholine (O-18:0_20:4) as potential mediators that reduce the likelihood of CD. Additionally, Triacylglycerol (48:0) was identified as a potential mediator that reduces the likelihood of IBD.

**Table 2:** The positive mediation effect of DR on IBD through circulating inflammatory proteins and plasma lipids.

Exposure	Mediator	Outcome	Beta (SE), P value			Mediating effect(95%CI)
			Exposure-Outcome	Mediator-Outcome	Exposure-Mediator	
DMP	Fibroblast growth factor 21	CD	-0.10484 (0.02054) P_value=3.34e-07	0.35156 (0.08875) P_value=7.46e-05	-0.036 (0.01259) P_value=4.26e-03	12.1% (8.6%-23.3%)
DMP	Phosphatidylcholine (20:4_0:0)	CD	-0.10484 (0.02054) P_value=3.34e-07	-0.13264 (0.031489) P_value=2.53e-05	0.04362 (0.01915) P_value=0.022709	5.5% (0–11.3%)
DR	Phosphatidylcholine (O-18:0_20:4)	CD	-0.139 (0.025) P_value=2.79e-08	-0.14466 (0.0656) P_value=0.02765	0.0387 (0.01934) P_value=0.0454	4.0% (0–9.5%)
DR	Triacylglycerol (48:0)	IBD	-0.08169 (0.02732) P_value=0.0027	-0.13789 (0.04293) P_value=0.00132	0.05259 (0.02246) P_value=0.01919	8.8% (0–19.7%)

CD, Crohn’s disease; IBD, Inflammatory bowel disease; DR, Diabetic retinopathy; DMP, Diabetic maculopathy; SE, Standard error; CI, Confidence interval.

## 4 Discussion

Numerous activities of the intestines are provided by the large amount of intestinal flora and its survival metabolism. Previous proposals have been proposed for the gut-kidney and gut-brain axes, and research on the relationship between the intestines and other tissues and organs has been conducted piecemeal. Based on our research, it was possible to link inflammation and metabolism to DR and IBD. Though these two illnesses appear to have different causes and manifestations in the human body, one must consider the significance of inflammatory variables and the integrity of human metabolism when considering the broader context. As there are no direct observational studies on DR and IBD, we cannot compare our results with those of real-world epidemiological surveys. We are investigating whether alternative mechanisms exist to bolster the notion of the gut-retina axis.

### 4.1 Causal effect of IBD on DR

Forward MR analysis has revealed a substantial negative correlation between UC and the risk of DR. Although our results indicated that circulating inflammatory proteins and plasma lipids did not mediate the causal relationship from IBD to DR in the mediation analysis, this relationship remained detectable. An increasing amount of research conclusively links iron to the pathological progression of retina disease, such as age-related macular degeneration (AMD) and DR. The inner blood-retina barrier (iBRB) protects retinal neural tissue from potentially hazardous compounds in circulation (24). Abnormal iron deposition disrupts iron homeostasis in retinal vascular endothelial cells (ECs), causing impairment of the blood-retinal barrier. Prior research has indicated that iron plays a pivotal role in the pathophysiology of DR by acting as a catalyst and facilitating reactive oxygen species (ROS)-induced dysfunction or death of ECs (25). Oxidative stress, characterized by a rise in hydroxyl radicals via the Fenton/Haber-Weiss mechanism, contributes significantly to retinal damage, affecting neurons, retinal pigment epithelial cells (RPEC), and retinal endothelial cells (REC) (26, 27). Iron also triggers canonical Wnt/β-catenin signaling in RPEC, which has been correlated with fibrosis, inflammation, and angiogenesis (28). The maintenance of retinal iron homeostasis is closely interconnected with systemic iron metabolism, relying on efficient iron absorption, transport, and utilization. Regardless of the structural integrity of the blood-retinal barrier, elevated serum iron levels can surpass regional processes that regulate retinal iron, potentially increasing the risk of age-related retinal diseases (29). In IBD, anemia, particularly iron deficiency anemia (IDA), is a common extraintestinal symptom. Increased intestinal mucosal inflammation in IBD leads to iron loss through gastrointestinal bleeding and impaired iron absorption, contributing to iron deficiency (30). The severity of intestinal inflammation has been shown to fluctuate with the extent of blood loss, which is more pronounced in UC than CD (31). Excessive blood loss exceeding dietary iron absorption can disrupt iron metabolism. Besides, hepcidin, a hepatocyte peptide hormone, regulates ferroportin activity on duodenal enterocytes. During inflammatory processes, cytokines like Interleukin-6 (IL-6) may induce hepcidin production, producing decreased iron transfer to plasma by altering ferroportin conformation (32). Systemic iron metabolism and storage are intricately linked to local iron homeostasis in the retina, necessitating further research to balance these factors effectively.

Furthermore, there’s an interesting substance called adiponectin worth thinking about. White adipose tissue secretes the secretory protein adiponectin, which has pro- and anti-inflammatory characteristics. It is important in insulin sensitivity, anti-inflammatory/anti-fibrotic processes, and anti-apoptotic mechanisms (33). Adiponectin has been proven to elevate insulin sensitivity and exert antidiabetic effects in mouse models. Research has demonstrated that adiponectin protects against pathological retinal microvessel formation by down-regulating TNF-α-mediated inflammatory responses (34). TNF-α is a cytokine involved in inflammation, insulin resistance, and diabetic vasculopathy. Adiponectin suppresses TNF-α production by inhibiting nuclear factor κB activity in macrophages and promotes macrophage clearance of apoptotic bodies, leading to decreased TNF-α levels (35, 36). Studies have shown that adiponectin levels tend to be greater in male and female UC patients compared to healthy controls, and elevated levels of adiponectin are observed in the serum of patients with CD (37, 38). Adiponectin levels vary in autoimmune and chronic inflammatory diseases such as rheumatoid arthritis (RA), chronic kidney disease (CKD), and IBD, exhibiting both pro- and anti-inflammatory properties relying on the specific tissue and signaling pathways involved (39).

Intestinal barrier dysfunction in UC can lead to decreased absorption of fluid, electrolyte, amino acid, fat, and carbohydrate, and disrupted intestinal motility. Abnormal results in tests assessing D-Xylose absorption, and reduced absorption of fat, folic acid, and amino acids, have been observed in individuals with UC (40). Systemic inflammation in UC and CD patients has been connected with lower levels of total and low-density lipoprotein (LDL) cholesterol compared with healthy individuals (41). Lowering cholesterol is considered an important aspect of diabetes treatment, and statins, lipid-lowering drugs, have been shown to bring down the risk of vision loss and the occurrence of hard exudates and microaneurysms in DR (42, 43). The formation of cholesterol crystals during the development of DR disrupts retinal lipid metabolism and contributes to persistent inflammation (44). Moreover, high-fat intake can induce hypothalamic inflammation, characterized by increased levels of circulating cytokines and free fatty acids, resulting in neuroinflammation and the release of proinflammatory cytokines such as TNF-α, IL-1β, and IL-6 (45, 46). This process also causes neurons to release fractalkine (CX3CL1), further intensifying inflammation by attracting peripheral monocytes to the hypothalamus (47). Hyperactivity of the sympathetic nervous system and hypothalamic inflammation are features of type 2 diabetes and metabolic syndrome (48). Hypothalamic inflammation and insulin resistance promote the development of DR by initiating inflammatory cascades and impairing pancreatic β-cell function and insulin production (49). In conclusion, a lower intake of lipids in UC may reduce the risk of triggering hypothalamic inflammation, thereby protecting against the development of DR.

### 4.2 Causal effect of DR on IBD

DR is a chronic inflammatory disease closely linked to lipid metabolism. MR studies suggest that phosphatidylcholine (PC) may mediate interactions between DR and CD. PC, a multifunctional phospholipid, is essential for cell membrane structure, fat metabolism, and cell signaling (50). Metabolomic analyses indicate some lower serum PC levels in NPDR and PDR groups compared to type 2 diabetes mellitus (T2DM) patients, except for PC (C34:4 and C36:6) which are lower in PDR than NPDR (51). Conversely, another study found elevated PC levels in DR patients compared to T2DM and non-DR patients (52), making the PC-DR relationship controversial and unclear. DR disrupts retinal lipid metabolism, which differs from plasma metabolism. The retina can synthesize docosahexaenoic acid (DHA, 22:6n3) from α-linolenic acid (18:3n3) and eicosapentaenoic acid (EPA, 20:5n3) (53). The relationship between DR and lipid compounds is influenced by medication and compensatory mechanisms, complicating cross-sectional studies. Similarly, IBD patients exhibit altered lipid profiles. In animal models, PC supplementation reduces DSS-induced colonic lesions and pro-inflammatory cytokines. PC is involved in tryptophan, arginine, proline, and purine metabolism, bile secretion, and vitamin absorption (54, 55). It helps treat IBD by regulating the intestinal barrier, reshaping gut microbiota, modulating macrophage polarization, and reducing inflammation. Dietary PC supplementation can alleviate intestinal inflammation and is emerging as a novel approach in clinical treatment (56, 57).

In studies of type 2 diabetes and insulin resistance, elevated FGF21 levels are considered a compensatory response to metabolic stress and are associated with both microvascular and macrovascular complications. However, the connection between FGF21 and DR is debated. Some researchers propose FGF21 as a biomarker for DR, noting higher blood levels in proliferative and non-proliferative DR patients compared to controls (58). On the other hand, other studies find no positive correlation, with some proposing a U-shaped relationship (59). They argue that individual conditions and medication history can influence the measurement of FGF21 in DR patients. Pemafibrate increases plasma and liver FGF21, which reduces retinal neovascularization, restoring retinal function, and lowering inflammatory markers (60). FGF21 also reduces hypoxia-induced neovascularization in proliferative DR (36). In animal models, FGF21 reduction exacerbates intestinal inflammation, while increased IL-22-mediated STAT3 activation maintains intestinal cell homeostasis (61). MR result suggests that reducing FGF21 can indirectly alleviate CD. Due to the controversial nature of FGF21’s relationship with DR and the impact of lifestyle and drugs on its levels, further investigation is necessary.

It is well-established that patients with DR and IBD often exhibit dyslipidemia. Cohort and case-control studies have shown that serum total cholesterol (TC), low-density lipoproteins (LDLs), and serum triglycerides (TGs) are significantly higher in patients with DME compared to non-DME patients (62). A predictive nomogram for DR risk in type 2 diabetes patients indicated that elevated TGs promote DR development (63). Cross-sectional studies have identified TGs as independent risk factors for DR, although observational studies only highlight associations between DR and TC without establishing causality (64). Our genetic analysis suggests that DR promotes increased TG levels, elucidating the interrelationship between these conditions. In the context of TGs and IBD, observational studies can determine whether IBD patients develop dyslipidemia but cannot assess whether dyslipidemia influences IBD progression. Data from the Korean National Health Insurance Service (2009–2016) showed an inverse relationship between serum TG levels and the incidence of UC, with lower TG levels linked to higher UC incidence (65). Our mediation analysis corroborates current observational studies and identifies causal relationships.

### 4.3 Limitations

It is important to consider the many restrictions that apply to our investigation. Firstly, DMP cannot be utilized as an outcome variable in forward MR to investigate the gut-retinal axis relationship because the number of SNPs in it is too limited. Secondly, since only people of European ancestry were included in the GWAS, it’s possible that the study’s conclusions cannot be applied to other ethnic groups. Thirdly, we selected the wide significance (P<5×10^−6^) as the threshold to get more SNPs in reverse MR, which could induce bias and false positive variants. Finally, the results of MR analysis and mediation analysis can point us in the right direction, but more clinical practice data are needed to supplement it. We are trying to study the signaling pathways associated with IBD and DR in future.

## 5 Conclusion

This MR study investigates the relationship between the gut-retinal axis in more detail. Although they manifest in different parts of the human body, IBD and DR share similar molecular processes and signaling pathways. Our MR and mediation analysis results indicate that DR and DME can reduce the incidence of CD. Additionally, DR can lower the incidence of IBD, while UC can reduce the incidence of DR. FGF21, PC and TG serve as mediators in these relationships. This research will help direct the development of specific medications and the management of these disorders.

## Data availability statement

The original contributions presented in the study are included in the article/**Supplementary Material**. Further inquiries can be directed to the corresponding authors.

## Author contributions

JYL: Conceptualization, Data curation, Formal analysis, Investigation, Methodology, Project administration, Software, Validation, Writing – original draft, Writing – review & editing. YC: Investigation, Methodology, Project administration, Validation, Visualization, Writing – review & editing. SG: Formal analysis, Investigation, Writing – review & editing. SS: Resources, Visualization, Writing – review & editing. HZ: Data curation, Resources, Writing – review & editing. JBL: Conceptualization, Project administration, Supervision, Writing – review & editing. SL: Conceptualization, Funding acquisition, Project administration, Supervision, Writing – review & editing.
